# Editorial: From vulnerability to vigor: innovative approaches in frailty and healthy aging

**DOI:** 10.3389/fmed.2026.1937906

**Published:** 2026-08-05

**Authors:** Pasquale Mone, Klara Komici, Leonardo Bencivenga, Giuseppe Rengo

**Affiliations:** 1 Department of Medicine and Health Sciences “Vincenzo Tiberio”, University of Molise, Campobasso, Italy; 2 Montevergine Clinic, GVM Care and Research, Mercogliano, Italy; 3 Department of Translational Medical Sciences, University of Naples Federico II, Naples, Italy; 4 Istituti Clinici Scientifici Maugeri - S.p.A. SB - Istituto di Ricerca e Cura a Carattere Scientifico (IRCCS) di Telese Terme, Telese Terme, Italy

**Keywords:** aging, comorbidities, frailty, functional decline, older adults

Frailty is a significant factor contributing to adverse events, particularly among older adults (1). The presence of various comorbidities can influence levels of frailty and the ability to maintain autonomy (2).

Frail older adults typically exhibit lower physical and cognitive functions, which leads to a higher risk of mortality, hospitalization, and complications during procedures (3). Therefore, a personalized treatment approach may be the most effective way to reverse frailty and enhance functional capabilities (4).

The present Research Topic has been titled: “*From vulnerability to vigor: innovative approaches in frailty and healthy aging*.”

In particular, Mariniello et al. found that frailty was associated with poorer lung function and greater physical impairment in patients with fibrotic interstitial lung diseases receiving antifibrotic therapy. Moreover, frail subjects required attention to find the right dosage.

The relationship between sarcopenia and frailty progression was addressed by Mu et al. whose longitudinal study in a Chinese cohort reinforced the close bidirectional link between muscle loss and functional decline.

Li et al. described the trajectory of post-operative frailty and cognitive function in elderly spinal surgery patients, while Montemurro et al. showed that loneliness in older adults affected not only cognitive performance but also the perception of one's own abilities.

Xin et al. found that family health and health literacy mediate the association between frailty and physical activity in preoperative patients with gastric cancer. Chen et al. surveyed nurses' knowledge, attitudes, and practices toward frailty syndrome, revealing gaps that may inform targeted training programs for healthcare professionals.

Intriguingly, Zhang et al. published a protocol demonstrating that nasoenteric tube insertion in frail older adults with chronic wounds is associated with improved nutritional

status, independent of underlying inflammatory states. In the context of infectious disease, De Vita et al. showed that a laboratory-based Frailty Index was a useful predictor of COVID-19 mortality.

Several studies focused on tools for the early identification and prediction of frailty trajectories. Silan et al. identified core adverse health outcomes suitable for frailty assessment using administrative data, offering a pragmatic approach for large-scale surveillance. Building on the need for predictive tools, Du et al. developed and validated nomograms to forecast frailty-worsening trajectories in older Chinese adults, while Lin et al. proposed the PRE-FRA model for predicting frailty risk in community-dwelling populations. At the population level, De Luca et al. reported preliminary results from the SUNFRAIL+ study, a multidimensional frailty-screening initiative across eight Italian regions, underscoring the feasibility of coordinated screening programs in real-world community settings.

Beyond clinical and functional scores, circulating biomarkers are increasingly investigated as complementary tools to capture the biological underpinnings of frailty and support earlier, more objective risk stratification (5).

Based on the published articles mentioned earlier, we can infer that frail patients often have multiple comorbidities that can negatively affect their physical and cognitive status. Physical therapy and rehabilitation can be treatment options for some frail older patients, but there is currently no approved medication specifically for frailty (6).

Many studies suggest using nutraceuticals to enhance the functional status of frail individuals (7). Studies recommend using sodium-glucose co-transporters 2 (SGLT2) inhibitors for patients with comorbidities such as diabetes, chronic kidney disease, and heart failure because their pleiotropic effects (8, 9).

Metformin is recommended for frail diabetic and prediabetic patients due to its anti-aging properties (10).

Thus, careful prevention is vital for promoting healthy aging. Frail patients should receive individualized treatment tailored to their comorbidities and functional status.
